# Tobacco craving and withdrawal symptoms in psychiatric patients during a motivational enhancement intervention based on a 26-hour smoking abstinence period

**DOI:** 10.18332/tpc/109785

**Published:** 2019-06-17

**Authors:** Ineke Keizer, Marianne Gex-Fabry, Patrice Croquette, Jean-Paul Humair, Aqal N. Khan

**Affiliations:** 1 Department of Mental Health and Psychiatry, Geneva University Hospitals (HUG), Geneva, Switzerland; 2 Faculty of Medicine, University of Geneva, Geneva, Switzerland; 3 Carrefour Addictions, CIPRET, Geneva, Switzerland

**Keywords:** psychiatry, craving, noncommunicable diseases, temporary smoking abstinence, motivational enhancement, tobacco withdrawal symptoms

## Abstract

**INTRODUCTION:**

In psychiatric patients, tobacco withdrawal symptoms are frequently seen as a barrier to smoking cessation; however, further studies are warranted in this specific population.

**METHODS:**

Patients receiving in- or out-patient mental health care participated in a motivational enhancement program including a 26-hour tobacco abstinence experience with professional support and optional nicotine replacement therapy. The study included 174 subjects, of whom 159 were evaluated 1 week before and at the end of the 26-hour abstinence period. Repeated assessments included the Minnesota Nicotine Tobacco Withdrawal Scale Revised (MNWS-R), two items of the Mood and Physical Symptoms Scale (MPSS) regarding craving, the State-Trait Anxiety Inventory (STAI-S), the Beck Depression Inventory (BDI-21), and the World Health Organization Well-Being Index (WHO-5).

**RESULTS:**

More than half the participants (52.3%) succeeded in 26-hour smoking abstinence. Craving was the most frequent MNWS-R withdrawal symptom (28.3% scored ≥3 on a 0–4 scale). Comparison of pre- and post-intervention data revealed significant improvements in 13 of 16 MNWS-R symptoms as well as craving (MPSS) and well-being, and significant decreases in anxiety and depression. Increasing MNWS-R craving scores and greater depression were both significantly associated with lower success in the 26-hour smoking abstinence period.

**CONCLUSIONS:**

The negative effects of tobacco withdrawal symptoms in psychiatric patients may be substantially overestimated. Participation in a supportive structured motivational intervention with a 26-hour smoking cessation experience was well tolerated and contributed to temporary improvements in mental state. Craving is an interesting symptom to evaluate during smoking cessation attempts.

## INTRODUCTION

Among addictions, smoking is the strongest and most difficult to stop, as demonstrated by cessation rates in randomized trials with placebo or no-treatment controls. Indeed, success rates at 6 months have been reported to be 47% for cocaine, 44% for opioids, 18% for alcohol, and only 8% for nicotine^1^. According to many smokers, craving is the major reason for failing or not attempting to stop. This concept has been described as ‘an abnormal desire or need to take a drug’^2^, including conscious and unconscious aspects. Although a large consensus supports the importance of craving, this construct remains complex to define and explain^3-5^. Craving is seen as a core symptom of addiction but also appears as a withdrawal effect in cessation. Craving is thus present in both smokers who repeatedly smoke another cigarette and smokers abstaining from smoking during a cessation attempt. In both cases, craving results from nicotine/tobacco deprivation, increasing shortly after abstinence (within hours or less) and lasting for several weeks or longer^6,7^. Craving during regular smoking or smoking cessation does not appear qualitatively different, but, during a cessation attempt with a longer abstinence period, discomfort increases, and a larger set of withdrawal symptoms occur together with the urge to smoke. Diagnostic criteria for nicotine withdrawal, according to DSM-IV^8^, include: irritability, frustration or anger; anxiety; difficulty concentrating; restlessness; decreased heart rate; increased appetite or weight gain; dysphoric or depressed mood; and insomnia. Controversy regarding whether craving should be considered a withdrawal symptom is illustrated by its inclusion in the criteria for nicotine dependence in the DSM-III edition, absence in the DSM-IV, and reintroduction in the DSM-V ‘tobacco use disorder’ entry. The different withdrawal symptoms are highly correlated, and factor analyses have suggested using a total score^9^, although combining elements specific to smoking (craving) with those less specific (e.g. anxiety, depression, irritability), or combining ‘psychological’ variables with somatic or physiological variables (hunger) may warrant further consideration^10,11^.

The high prevalence of smoking in psychiatric patients is well established, the odds that a patient with bipolar disorder will become a current smoker are 3.5 times higher than those from people from the worldwide general population^12^, and are even higher for patients with schizophrenia. Rates of 36.1%^13^, 53%^14^ or 67%^15^ are described in patients with schizophrenia or non-affective psychosis. Although this population accounts for a growing proportion of all smokers^13,16^, barriers prevent optimal interventions^17,18^. The assumption that smoking cessation may be a risk factor for increased psychopathology in psychiatric patients is widespread^19,20^, although most recent studies emphasize the benefits of cessation^21^ and the self-medication hypothesis of smoking is questioned^22^. In our clinical practice, we frequently encounter patients reporting that they are unable to stop smoking because they fear being unable to tolerate craving symptoms, or missing smoking’s benefits of anxiety or stress relief. Evaluation of nicotine withdrawal symptoms in psychiatric patients has encountered methodological difficulties, owing to the overlap of these symptoms with those common in several mental health disorders (e.g. anxiety, depression, and sleep disturbance)^23^. In a sample of smokers with attention-deficit hyperactivity disorder (ADHD) assessed before any cessation attempt, nicotine withdrawal symptoms (difficulty concentrating, restlessness or impatience, and anxiety or nervousness) positively correlated with current ADHD symptoms. These symptoms were significantly more severe in smokers with a history of ADHD than in smokers without ADHD, thus illustrating the difficulty in interpreting symptoms potentially influenced by both mental disorders and tobacco withdrawal^24,25^. Psychiatric patients have frequently been excluded from research on smoking cessation and development of instruments in this domain, thus calling into question the generalizability of results to populations with mental health conditions. Therefore, careful documentation of nicotine deprivation symptoms in relation to psychiatric symptoms remains a challenge in these populations, particularly regarding tobacco use and cessation.

The present study investigated nicotine withdrawal symptoms in a sample of smokers presenting with psychiatric disorders and receiving mental health care from in- or out-patient public services. They participated in a short motivational enhancement program based on a 26-hour smoking abstinence period. The first aim was to measure the prevalence of withdrawal symptoms and to study changes before and during the intervention in this specific population, by using the Minnesota Nicotine Withdrawal Scale Revised (MNWS-R or MTWS-R)^26,27^. The second aim was to analyze the factor structure of the MNWS-R and correlations with specific instruments measuring anxiety, depression, and craving. The third aim was to investigate aggravation or improvement in withdrawal symptoms during short-term abstinence and their association with successful or unsuccessful smoking abstinence.

## METHODS

### Setting and intervention

Participants were patients receiving in- and out-patient services at a public psychiatric institution, the Department of Mental Health and Psychiatry of the Geneva University Hospitals. The in-patient setting comprised seven acute care or rehabilitation units, and the out-patient setting comprised four ambulatory sectors providing intensive day care and consultation services. An indoor smoking ban applied to all settings, but patients whose condition allowed them to go outdoors were able to smoke there without restrictions. An intervention was designed to motivate smoking cessation in psychiatric patients. This multicomponent intervention (called ‘Journée parenthèse’ or ‘Day off’) began on Thursday 8:30 am and ended on Friday 10:30 am, and was the same for in- and out-patients. It was based on a 26-hour tobacco abstinence period and a structured day program during which participants benefitted from group support and attended thermal baths, restaurants, and tobacco information sessions, and participated in entertaining activities. Nicotine replacement therapy (NRT) was optional but strongly recommended. The aim of the program was to induce a strong positive experience and personal involvement, in order to mobilize motivational resources initiating a smoke-cessation process, according to the theory that positive outcome experiences influence motivational and intention forming elements, which may lead to behavioral changes^28^. The intervention can be considered successful when participants have experienced a positive outcome related to smoking during the ‘Day off’ intervention. A detailed description of this motivational enhancement program has been published elsewhere^29^.

### Recruitment

Staff from all units of the institution received information about the intervention. Patients were contacted by their care providers and invited to participate. Subjects could be enrolled if they had smoked recently (regardless of frequency of consumption) and if their psychiatric symptoms and clinical state were stable enough to integrate group participation and multiple activities. Further, they were required to be willing to participate and to commit to abstain from smoking during 26 hours. Repeated participation was possible, but only data related to the first enrolment were used in this study. The exclusion criteria were cognitive impairment or insufficient French fluency. The study protocol was approved by the ethics committee of the Geneva University Hospitals. All participants provided written informed consent before entering the study.

Among 389 participants enrolled in the program over a 7-year period, 174 were included in the study (115 inpatients and 59 outpatients). The reasons for non-inclusion were cognitive impairment, language barriers, or lack of informed consent (n=116); repeated participation in the program (n=48), absence on the intervention day, e.g. because of worsened clinical condition or discharge from the hospital (n=43); or leaving the program during the first hours (n=4). Four other participants were excluded because they had recently stopped smoking but participated to reinforce their motivation. Among 174 subjects in the study, 159 were reassessed after the 26-hour intervention. Fifteen patients were not reassessed for various reasons (staying asleep, having another priority appointment, and lack of motivation, possibly in relation to resumed smoking).

### Study design and instruments

Participants were evaluated on two occasions. The first interview took place during the week preceding the intervention: sociodemographic and diagnostic information was collected, and psychological and tobacco-related instruments were used, as described below. The second evaluation was performed with the same instruments, after the intervention and 26-hour smoking abstinence period (Friday morning).

Withdrawal symptoms were measured with the MNWS-R/MTWS-R scale^26,27,30^, which includes 16 items (anger or frustration, anxiety, depression, craving, difficulty concentrating, increased appetite, insomnia, restlessness, impatience, constipation, dizziness, coughing, dreaming or nightmares, nausea, sore throat, and hot flushes or shivers) rated on a 4-point Likert scale (0=not present to 3=severe). Craving was also measured with the total score of two items of the Mood and Physical Symptoms Scale^31^ (MPSS): ‘How much of the time have you felt the urge to smoke today?’ and ‘How strong have the urges been today?’ (from 0=not at all/no urges to 5=all the time/extremely strong). MNWS-R and MPSS were proposed at pre-evaluation for collection of baseline data, even if patients were smoking ‘as usual’. The Heaviness of Smoking Index^32,33^ (HSI) was calculated on the basis of two questions of the Fagerström Test for Nicotine Dependence (FTND): ‘How many cigarettes a day do you smoke?’ (answers in four categories) and ‘How soon after you wake up do you smoke?’ (four categories). Readiness to stop smoking was evaluated with Biener’s Contemplation Ladder^34^. Anxiety and depression were measured with the State-Trait Anxiety Inventory, state part^35^ (STAI-S) and the Beck Depression Inventory^36^ (BDI-21), respectively. Well-being was evaluated with the WHO-5 index^37,38^, with raw scores with a cut-off point at 11 used in psychiatric settings, to assess well-being^39^. Exhaled carbon monoxide (CO) was measured with a piCO+ Smokerlyzer (Bedfont, UK) at the start and end of the first intervention day (Thursday 9:15 am and 6:15 pm) and on the next morning (Friday 10:15 am).

### Statistics

Categorical variables were described with frequency tables (n, %). Ordinal and continuous variables were described with medians (ranges). Differences between independent groups were tested with Fisher’s exact test for categorical variables and the Mann-Whitney U-test for ordinal and continuous variables. Change over time was tested with the non-parametric sign test (two time points) or the Friedman analysis of variance (more than two time points). Spearman’s rank correlation coefficient (ρ) was used to test associations between dimensions. The internal structure of the 16-item MNWS-R was explored with principal component analysis with oblique rotation (promax method) to allow for possible correlations between components. The number of retained components was determined according to the following criteria: 1) Kaiser’s criterion, i.e. retaining components with eigenvalues ≥1; 2) the scree plot method, i.e. retaining components in the steep part of the graph; and 3) percentage explained variance. Items with loadings of at least 0.50 were considered to be part of a given factor, provided that there was no overlap with other factors (cross-loadings <0.50). Statistics were computed in SPSS version 22 (IBM Corporation, Armonk, NY). All tests were two-tailed, with the significance threshold at 0.05.

## RESULTS

### Sociodemographic, clinical, and smoking characteristics at pre-intervention

Characteristics of the study sample are summarized in Table 1. The most frequent primary diagnoses were mood disorders (40.2%) and psychotic disorders (41.4%). According to the anxiety (STAI-S) and depression rating scales (BDI-21), 55.0% had moderate to very high anxiety scores, and 40.5% had moderate or severe depression scores.

**Table 1:** Sociodemographic, clinical and smoking characteristics at pre-intervention (N=174)

		*n*	*%*	
**Sociodemographic characteristics**				
Gender	Male	100	57.5	
Age [median, min–max (range)]		35.5	17–64 (47)	
Education (N=170)	Compulsory or less	69	40.6	
	Intermediate	87	51.2	
	Higher	14	8.2	
Financial condition (N=169)	Disability pension or social aid	107	63.3	
	Worker or student (no long-lasting disability)	62	36.7	
**Clinical characteristics**				
Primary diagnosis	Psychoactive substance disorders	10	5.7	
	Psychotic disorders	72	41.4	
	Mood disorders	70	40.2	
	Other disorders	22	12.6	
Comorbidity (ICD-10 psychiatric diagnoses)	1 diagnosis	79	45.4	
	2 diagnoses	56	32.2	
	3 or more diagnoses	39	22.4	
Anxiety (STAI-S) (N=151)	Very low (<36)	36	23.8
	Low (36–45)	32	21.2
	Medium (46–55)	46	30.5
	High (56–65)	26	17.2
	Very high (>65)	11	7.3
Depression (BDI-21) (N=153)	Absent (<10)	33	21.6
	Mild (10–19)	58	37.9
	Moderate (20–29)	41	26.8
	Severe (≥30)	21	13.7
Well-being (WHO-5) (N=163)	Positive well-being (≥ 11)	96	58.9
	Low well-being (<11)	67	41.1
**Smoking characteristics**			
Cigarettes per day (N=173)	≤10	56	32.4
	11–20	59	34.1
	21–30	38	22.0
	≥31	20	11.6
Heaviness of smoking index (HSI) [median, min–max (range)] (N=171)		4.0	1–7 (6)
Carbon monoxide [median, min–max (range)] (N=171)	ppm	21.0	1–100 (99)
Quit attempt in past 6 months (N=169)	Yes	41	24.3
	No	128	75.7
Stage of change	Pre-contemplation	90	51.7
	Other stage	84	48.3
Preparation to quit (Biener) [median, min–max (range)] (N=173)		7.0	0–10 (10)
Craving (MPSS subscale) [median, min–max (range)] (N=173)		5.0	0–10 (10)

One-third were heavy smokers (>20 cigarettes/day), and the median CO was 21 ppm (range 1–100). More than half (51.7%) were in the pre-contemplation stage, and 75.7% had not made a cessation attempt in the previous 6 months. The nicotine dependence (HSI score) did not significantly correlate with anxiety (ρ=0.05, p=0.56) or depression (ρ=0.02, p=0.85), but was significantly associated with decreased well-being (WHO-5; ρ=-0.15, p=0.05) and increased craving (MPSS; ρ=0.47, p<0.001). The HSI score was not significantly different for NRT users/no-users (n=130, median=4 vs n=19, median=4; Mann-Whitney U-test p=0.10). The number of cigarettes smoked per day might have been related to NRT use (medians of 20 for users and 10 for the no-NRT subgroup; Mann-Whitney U-test p=0.05).

Comparison of in- and out-patients showed no significant differences for any diagnostic or smoking characteristics; these groups differed only in age (inpatients were younger; Mann-Whitney U-test p<0.001) and sex (inpatients included more males; Fisher’s exact test p=0.04) but not in other sociodemographic data.

In Table 1, some data are missing because of non-reliable answers (i.e. financial condition or past cessation attempts) but mainly because of concentration difficulties related to mental health conditions when longer questionnaires were used (i.e. STAI anxiety or BDI depression).

### Outcomes of the 26-hour smoking abstinence intervention

NRT was largely used during the ‘Day off’ intervention: 86.8% of participants took fast and/or slow acting products, and the dosage was adapted to the usual cigarette consumption according to standard medical recommendations. CO decreased significantly during the intervention (median 18 ppm at 9:15 at the start of the intervention; 7 ppm at 18:15 at the end of the first day; and 6 ppm at 10:15 on the next day; Friedman test p<0.001). At the end of the intervention, the median CO was 4 ppm for those succeeding in abstinence and 11 ppm for those not succeeding.

Of 174 participants, 8.6% (n=15) smoked one or several cigarettes during the intervention day, and 9 out of 10 (89.7%, n=156) did not smoke during the intervention day (9-hour abstinence). Smoking abstinence was further maintained during the 26-hour period after participants spent the evening and night in an uncontrolled environment (the usual living place for outpatients or a hospital ward for inpatients) in 52.3% (n=91) of participants. Information was missing for three participants (e.g. CO value inconsistent with self-reported data). Participants who did and did not succeed in 26-hour abstinence did not differ in baseline anxiety, depression, well-being, and craving, but nicotine dependence (HSI) was significantly lower among those who succeeded (median 4.0 vs 5.0, Mann-Whitney U-test p=0.03).

### Withdrawal symptoms during the 26-hour abstinence intervention

Table 2 shows the percentages of participants with symptoms either absent (score 0) or marked/severe (scores 3 or 4) according to the MNWS-R, as reported after the 26-hour abstinence intervention. Craving was the most frequent symptom (28.8% of participants with scores 3 or 4), whereas 15 to 22% reported elevated levels of anxiety, depressed mood, difficulty concentrating, increased appetite, restlessness, and impatience. Five withdrawal symptoms (constipation, dizziness, nausea, sore throat, hot flushes, or shivers) were absent in more than 50% of subjects. Sleep difficulties and nightmares were present in approximately half of the subjects and were marked in 20–24% of the subjects. Median scores are provided in Table 3a.

**Table 2:** Results on the Minnesota Withdrawal Scale (MNWS-R) during abstinence trial (N=152)

*MNWS-R items*	*Absent^a^*	*Marked or severe^b^*	*PCA factor loadings^c^*		
	*n (%)*	*n (%)*	*Factor 1*	*Factor 2*	*Factor 3*
Angry, irritable, frustrated	68 (44.7)	19 (12.5)	**0.79**	0.27	0.38
Anxious, nervous	40 (26.3)	25 (16.4)	**0.80**	0.48	0.15
Depressed mood, sad	59 (38.8)	26 (17.1)	**0.66**	0.49	0.19
Desire or craving to smoke	34 (22.4)	43 (28.3)	**0.68**	0.27	0.18
Difficulty concentrating	50 (32.9)	22 (14.4)	0.47	**0.67**	0.19
Increased appetite, hungry, weight gain	57 (37.5)	30 (19.7)	0.20	0.35	**0.55**
Insomnia, sleep problems, awakening at night	75 (49.3)	34 (22.4)	0.34	**0.63**	0.30
Restless	52 (34.2)	25 (16.5)	**0.84**	0.45	0.28
Impatient	39 (25.7)	32 (21.1)	**0.75**	0.46	0.16
Constipation	105 (67.3)	16 (10.3)	0.24	**0.63**	0.17
Dizziness	103 (67.8)	16 (6.6)	0.44	**0.78**	0.29
Coughing	75 (49.3)	18 (11.9)	0.29	0.14	**0.78**
Dreaming or nightmares	76 (50.0)	31 (20.4)	0.22	0.41	0.45
Nausea	115 (75.7)	5 (3.3)	0.15	0.38	**0.67**
Sore throat	99 (65.1)	12 (7.9)	0.22	0.21	**0.80**
Hot flushes, shivers	91 (59.9)	19 (12.5)	0.40	**0.74**	0.31

a score=0.

b score=3 or 4.

c bold: loadings >0.50.

**Table 3a:** Withdrawal symptoms before the intervention and after 26-hour abstinence trial (N=174)

	*Pre intervention*	*End of intervention*	*Improved^a^*		*Worsened^b^*		*Sign test*
	*Median*	*Median*	*n^e^*	*%*	*n^e^*	*%*	*p*
**Withdrawal symptoms (MNWS-R)** (range 0–4)							
Angry, irritable, frustrated (n=153)^c^	1.0	1.0	65	42.5	25	16.3	<0.001
Anxious, nervous (n=153)	2.0	1.0	96	62.7	12	7.8	<0.001
Depressed mood, sad (n=151)	2.0	1.0	76	50.3	23	15.2	<0.001
Desire or craving to smoke (n=152)	2.0	2.0	70	46.1	32	21.1	<0.001
Difficulty concentrating (n=153)	2.0	1.0	77	50.3	24	15.7	<0.001
Increased appetite, hungry, weight gain (n=151)	1.0	1.0	49	32.5	48	31.8	1.0
Insomnia, sleep problems, awakening at night (n=153)	1.5	1.0	67	43.8	29	19.0	<0.001
Restless (n=153)	2.0	1.0	72	47.1	31	20.3	<0.001
Impatient (n=153)	2.0	1.0	69	45.1	37	24.2	0.003
Constipation (n=163)	0.0	0.0	27	16.6	20	12.3	0.38
Dizziness (n=153)	1.0	0.0	56	36.6	23	15.0	<0.001
Coughing (n=152)	1.0	1.0	53	34.9	28	18.4	0.008
Dreaming or nightmares (n=152)	1.0	1.0	64	42.1	27	17.8	<0.001
Nausea (n=153)	0.0	0.0	35	22.9	22	14.4	0.11
Sore throat (n=152)	0.0	0.0	37	24.3	27	17.8	0.26
Hot flushes, shivers (n=152)	1.0	0.0	57	37.5	22	14.5	<0.001
**Craving**							
MPSS (observed range 0–10) (n=157)	5.0	4.0	88	56.1	32	20.4	<0.001
**Anxiety**							
STAI-S (observed range 20–77) (n=120)	47.0	37.0	88	73.3	26	21.7	<0.001
**Depression**							
BDI-21 (observed range 0–52) (n=128)	17.0	13.0	89	69.5	31	24.2	<0.001
**Well-being**							
WHO-5 (observed range 0–25) (n=142)	12.0	17.0	103	72.5	24	16.8	<0.001

a Score decreased over time, except for WHO-5 which increased.

b Score increased over time, except for WHO-5 which decreased.

c Completed at both pre-intervention and end of intervention.

**Table 3b:** Withdrawal symptoms before the intervention and after 26-hour abstinence trial according to NRT (N=174)

	*Improved^a^*				*Worsened^b^*				*Sign test^d^*	
	*NRT*	*No NRT*	*NRT*	*NO NRT*	*NRT*	*No NRT*	*NRT*	*No NRT*	*NRT*	*No NRT*
	*n^c^*	*%*	*n^c^*	*%*	*n^c^*	*%*	*n^c^*	*%*	*p*	*p*
**Withdrawal symptoms (MNWS-R)** (range 0–4)										
Angry, irritable, frustrated (n=153)^c^	55	41.1	10	52.6	23	17.2	2	10.5	**<0.001**	**0.04**
Anxious, nervous (n=153)	86	64.2	10	52.6	11	8.2	1	5.3	**<0.001**	**0.01**
Depressed mood, sad (n=151)	70	53.0	6	31.6	20	15.2	3	15.8	**<0.001**	0.51
Desire or craving to smoke (n=152)	64	48.1	6	31.6	25	18.8	7	36.8	**<0.001**	1.0
Difficulty concentrating (n=153)	72	53.7	5	26.3	20	14.9	4	21.1	**<0.001**	1.0
Increased appetite, hungry, weight gain (n=151)	43	32.6	6	31.6	47	35.6	6	31.6	1.0	1.0
Insomnia, sleep problems, awakening at night (n=153)	62	46.3	5	26.3	28	20.9	1	5.3	**0.001**	0.22
Restless (n=153)	68	50.8	4	21.1	25	18.7	6	31.6	**<0.001**	0.75
Impatient (n=153)	65	48.5	4	21.1	33	24.6	4	21.1	**0.002**	1.0
Constipation (n=163)	25	18.8	2	10.5	19	14.3	1	5.3	0.45	1.0
Dizziness (n=153)	52	38.8	4	21.1	19	14.2	4	21.1	**<0.001**	0.22
Coughing (n=152)	48	36.1	5	26.3	26	19.6	2	10.5	**0.02**	0.45
Dreaming or nightmares (n=152)	59	44.4	5	26.3	24	18.1	3	15.8	**<0.001**	0.73
Nausea (n=153)	32	23.9	3	15.8	18	13.4	4	21.1	0.07	1.0
Sore throat (n=152)	32	24.1	5	26.3	25	18.8	2	10.5	0.43	0.45
Hot flushes, shivers (n=152)	52	39.1	5	26.3	22	16.5	0	0	**0.001**	0.06
**Craving**										
MPSS (observed range 0–10) (n=157)	79	57.2	8	47.4	26	18.8	6	31.6	**<0.001**	0.61
**Anxiety**										
STAI-S (observed range 20–77) (n=120)	79	73.8	9	69.2	23	21.5	3	23.1	**<0.001**	0.15
**Depression**										
BDI-21 (observed range 0–52) (n=128)	80	70.2	9	64.3	28	24.6	3	21.4	**<0.001**	0.15
**Well-being**										
WHO-5 (observed range 0–25) (n=142)	93	73.8	10	62.5	21	16.7	3	18.8	**<0.001**	0.09

a Score decreased over time, except for WHO-5 which increased.

b Score increased over time, except for WHO-5 which decreased.

c Completed at both pre-intervention and end of intervention.

d Sign test, binomial distribution used.

Significant correlations were observed between the MNWS-R item ‘anxious, nervous’ and the STAI-S anxiety score (ρ=0.55, p<0.001); the item ‘depressed mood, sad’ and the BDI-21 depression score (ρ=0.45, p<0.001); and the item ‘desire or craving to smoke’ and the MPSS craving score (ρ=0.69, p<0.001).

Further inspection of MNWS-R items according to use of NRT resulted in very small subgroups, thus limiting the possibility and power of statistical analyses. In Table 2, only the item ‘craving’ had sufficient data for minimal analysis (expected cell counts >5) and showed no significant differences according to use of NRT and the item craving absent vs marked or severe (n=77; Fisher’s exact test p=0.38).

Principal component analysis (Table 2) was used to investigate whether MNWS-R symptoms might belong to one or several constructs: whereas Kaiser’s criterion suggested a five-component structure, the scree plot led to retention of three components (eigenvalues 5.3, 1.8, and 1.3), which explained 52.6% of the total variance. The first factor was mainly contributed by ‘psychological’ aspects, such as craving, anxiety, depression, irritability, restlessness, and impatience. The factor scores were significantly associated with MPSS (ρ=0.55, p<0.001), WHO-5 (ρ=-0.67, p<0.001), STAI-S (ρ=0.62, p<0.001), and BDI-21 (ρ=0.53, p<0.001). The second factor included difficulty concentrating, sleep problems, constipation, dizziness, and hot flushes or shivers. The factor scores similarly correlated with MPSS (ρ=0.23, p=0.005), WHO-5 (ρ=-0.38, p<0.001), STAI-S (ρ=0.52, p<0.001), and BDI-21 (ρ=0.43, p<0.001). The third factor included increased appetite, coughing, nausea, and sore throat. The factor scores correlated with STAI-S (ρ=0.20, p=0.03) and BDI-21 (ρ=0.27, p=0.002), but not MPSS (ρ=0.05, p=0.55) and WHO-5 (ρ=-0.09, p=0.31).

### Changes between pre- and post-intervention (at 26-hour) assessments

As indicated in Table 3a, 13 of 16 items in the MNWS-R significantly improved after the intervention, including the following MNWS basic elements: irritability, anxiety, depressed mood, difficulty concentrating, restlessness, and craving. The only symptoms that did not change significantly were appetite, nausea, and sore throat. Anxiety (STAI-S), depression (BDI-21), well-being (WHO-5), and craving (MPSS) similarly improved between pre-intervention and 26-hour assessments (sign tests, all p<0.001).

Further inspection of changes (improvement or aggravation) according to NRT had very limited statistical power to detect differences in the small no-NRT subgroup, because most subjects (86.8%) used some form of NRT. As shown in Table 3b, most items in the NRT subgroup improved significantly more than worsened. For the no-NRT subgroup, improvement was detected for anger and anxiety in the MNWS-R items but not for craving in the MNWS-R or MPSS scores. However, 48.1% with NRT improved compared with 31.6% without, and 18.8% worsened with NRT compared with 36.8% without.

### Outcomes of 26-hour smoking abstinence according to changes in withdrawal symptoms

In Table 4, the proportions of successful or unsuccessful 26-hour abstinence are compared for participants whose withdrawal symptoms worsened or improved during the intervention. The success rate was significantly lower among participants who reported worsening on the MNWS-R item ‘craving’ than among those who reported improving or remaining stable (37.0% vs 66.0%, Fisher’s exact test p=0.008). Among participants who reported increased coughing, 80.8% were successful, compared to 54.2% of those without increased coughing (p=0.02). For all other MNWS-R items, improvement or aggravation was not significantly associated with the outcome of the 26-hour abstinence intervention. The proportion of success was also significantly lower with worsening on the MPSS craving subscale (41.4% vs 64.2%, p=0.03) and the BDI-21 (increasing depression score) (41.4% vs 83.8%, p=0.02).

**Table 4:** Comparison of patients whose symptoms worsened or improved during the intervention with respect to successful 26-hour abstinence (N=174)

	*Symptoms worsened during 26-h abstinence*				*Symptoms were stable or improved during 26-h abstinence*				*Fisher’s test*
	*Successful abstinence*		*Unsuccessful abstinence*		*Successful abstinence*		*Unsuccessful abstinence*		
	*n*	*%*	*n*	*%*	*n*	*%*	*n*	*%*	*p*
**Withdrawal symptoms (MNWS-R)**									
Angry, irritable, frustrated (n=134)	9	42.9	12	57.1	71	62.8	42	37.2	0.10
Anxious, nervous (n=134)	4	44.4	5	55.6	76	60.8	49	39.2	0.48
Depressed mood, sad (n=132)	10	47.6	11	52.4	69	62.2	42	37.8	0.23
Desire or craving to smoke (n=133)	10	37.0	17	63.0	70	66.0	36	34.0	**0.008**
Difficulty concentrating (n=134)	10	52.6	9	47.4	70	60.9	45	39.1	0.62
Increased appetite, hungry, weight gain (n=132)	26	60.5	17	39.5	54	60.7	35	39.3	1.00
Insomnia, sleep problems, awakening at night (n=134)	13	52.0	12	48.0	67	61.5	42	38.5	0.50
Restless (n=134)	14	48.3	15	51.7	66	62.9	39	37.1	0.20
Impatient (n=134)	15	48.4	16	51.6	65	63.1	38	36.9	0.15
Constipation (n=133)	12	66.7	6	33.3	68	59.1	47	40.9	0.61
Dizziness (n=134)	10	55.6	8	44.4	70	60.3	46	39.7	0.80
Coughing (n=133)	21	80.8	5	19.2	58	54.2	49	45.8	**0.02**
Dreaming or nightmares (n=133)	17	70.8	7	29.2	63	57.8	46	42.2	0.26
Nausea (n=134)	10	50.0	10	50.0	70	61.4	44	38.6	0.46
Sore throat (n=133)	15	62.5	9	37.5	64	58.7	45	41.3	0.82
Hot flushes, shivers (n=133)	12	60.0	8	40.0	68	60.2	45	39.8	1.00
**Craving**									
MPSS (n=138)	12	41.4	17	58.6	70	64.2	39	35.8	**0.03**
**Anxiety**									
STAI-S (n=113)	11	45.8	13	54.2	60	67.4	29	32.6	0.06
**Depression**									
BDI-21 (n=120)	12	41.4	17	58.6	62	83.8	29	31.9	**0.02**
**Well-being**									
WHO-5 (n=133)	21	55.3	17	44.7	59	62.1	36	37.9	0.56

## DISCUSSION

In the present sample of patients with severe mental health disorders, the prevalence of moderate to severe anxiety (STAI-S) and depression (BDI-21) was >40% at pre-intervention. ‘Withdrawal symptoms’ (MNWS-R) were already observable at pre-intervention, with median scores of 2 for anxiety, depression, craving, difficulty concentrating, restlessness, and impatience. This observation may raise the question of whether withdrawal symptoms might already have been present during continued smoking. In any case, the results confirm the methodological difficulties in studying withdrawal effects in psychiatric patients, because these symptoms are also frequent in psychiatric conditions. During the 26-hour abstinence trial, craving appeared to be a more prominent symptom rated higher than other symptoms on the MNWS-R scale, also among patients with confounding symptoms due to mental illness. It was a severe symptom in 28.8% of participants. This finding is consistent with observations showing that abstinence effects are largest for craving^40^. A factorial analysis of the MNWS-R showed that craving belonged to a factor that included anxiety, depression, irritability, restlessness, and impatience, thus underlining the difficulty in disentangling it from other symptoms. Significant correlations of all three withdrawal components with anxiety and depression scores further confirmed the difficulty in interpreting such factors. However, conceptually, craving was the only item specifically related to smoking, thus suggesting that although it is associated with other aspects of withdrawal, it remains central in evaluating smoking abstinence. Withdrawal seems related to a non-specific reaction to the stress of abstinence, whereas craving could be considered the ‘tip of the iceberg’ in the withdrawal syndrome.

Unexpectedly, analysis of changes before and after intervention showed that patients’ traditional withdrawal symptoms clearly improved during abstinence, in contrast with descriptions of increased discomfort after smoking cessation. For most withdrawal symptoms, including craving, as well as for psychiatric symptoms (anxiety, depression) and well-being, improvement between pre- and post-intervention was significantly more common than aggravation.

Understanding the observed improvement of withdrawal symptoms after abstinence is challenging, and limitations to study this topic include the following:

- This intervention includes smokers not searching counseling support for cessation, in contrast to most studies of smoking-cessation. Subjects seeking assistance in cessation might have more acute withdrawal symptoms.
- The diversity in individual smoking profiles was high, because the present intervention was designed to include all smokers according to a broad definition. Although mean tobacco use was important, 32.4% of participants smoked ≤10 cigarettes per day. Hence, one-third of smokers probably did not experience important tobacco withdrawal symptoms and related discomfort.
- The sample also included one-third of heavy smokers (33.6% smoked >20 cigarettes per day) for whom high anticipatory anxiety at pre-intervention might have contributed to the global decrease in negative affect during the intervention.

Patients with mental health disorders might also have higher anxiety or be less able to manage negative affect. We observed that heavy smokers or those smoking for decades sometimes showed substantial anticipatory uncertainty about their ability to not smoke for 26 hours. The literature has reported anticipatory and psychological reactions to cessation and, for some smokers, increased negative affect even before cessation^41^, and unfounded fears about having a strong desire to smoke after cessation have been described in non-psychiatric samples^42^.

- All subjects in our sample received psychiatric treatment including pharmacotherapy, which may have blunted the increase in withdrawal symptoms during smoking abstinence. Improvements associated with mental health treatment may also have occurred between pre- and post-intervention; however, such effects probably remained marginal, given the short period.
- Finally, components of the intervention itself should be mentioned: having a busy and pleasant schedule, being out of one’s usual environment, the need for increased attentional resources and being distracted from everyday functioning, engaging in light physical exercise (thermal baths), receiving group support from other participants and professionals, and experiencing the positive psychological effects of a successful intervention might have contributed to decreased withdrawal symptoms.

Our study results provide deeper understanding of the relationship between short-term 26-hour smoking abstinence success and withdrawal symptoms. Among all the MNWS-R items, only craving and coughing, as well as craving on the MPSS subscale and depression on the clinical depression scale (BDI-21), were significantly associated with successful abstinence.

Craving evolved differently between successful and unsuccessful abstinence, clearly decreasing during the intervention for abstainers (more than half the sample), whereas it remained high in those who relapsed. The significant association between stronger craving and a failure to maintain temporary abstinence confirms that craving can be considered a risk factor for smoking relapse after cessation, but this association would mainly apply to the subgroup with unsuccessful cessation attempts. Intense craving during a quit attempt might be a sign of unsuccessful abstinence. The finding that successful abstainers showed lower craving during abstinence indicates that increased craving is not necessarily associated with interruption of nicotine intake from cigarettes. Such considerations were discussed more than 30 years ago, as lighter craving was observed in subjects totally abstaining from smoking than in those continuing smoking^43^. Other studies confirmed that post-cessation craving is a better indicator of successful abstinence than pre-cessation craving^44^, and that exacerbation of symptoms of depression is more strongly associated with unsuccessful cessation attempts than successful abstinence^45,46^. Differences in craving between successful and unsuccessful abstainers shortly after cessation has further been described^41^, thus indicating that smokers’ paths diverge more after than before a cessation attempt. In particular, craving is more tightly linked with reports of recent smoking during a cessation attempt than any other symptom. Thus, to predict success, craving should be assessed post-cessation. Craving is interesting as relapse indicator only in the case of abstinence; consequently, craving may be a useful indicator of cessation success during abstinence, but it may be conceptualized as a core symptom of smoking forming part of a ‘chronic withdrawal cycle’^45^.

In our data, other significant differences between successful and unsuccessful 26-hour smoking abstinence trials involved ‘coughing’ in the MNWS-R, which was more pronounced for successful abstainers. Coughing is a common and normal reaction after smoking cessation^47^, owing to the reactivation of airway clearance by the mucociliary system. Cigarette smokers have diminished cough-reflex sensitivity compared to non-smokers, eventually related with a centrally mediated antitussive effect of nicotine^48^, and intensity of smoking is known to be associated with impairment of nasal mucociliary clearance. Smoking cessation rapidly induces improvements in mucociliary clearance^49^ and reverses inhibition of cough-reflex sensitivity^50^. Our data suggest that this could occur very rapidly, already after 26 hours of smoking abstinence, for participants who did not smoke.

Our study also confirmed that depression, assessed on a validated clinical scale, improved more for abstainers, as evaluated during a short-term period. In the longer term, improvement in mental health conditions upon smoking cessation relies on a growing body of research indicating positive experiences with psychiatric patients stopping smoking and improvements in psychological functioning for quitters even in groups with severe psychiatric conditions^45^. The literature indicates that lower stress after smoking cessation is a robust and empirical phenomenon^51^ and that abstinence is not associated with increased affective distress, even in smokers with depressive disorders^45^. Our results pertain to only a short 26-hour abstinence trial, but they clearly illustrate that even severely dependent smokers, in a favorable context with NRT, mostly did not present with severe withdrawal effects or increased negative affect when smoking was interrupted. Most subjects used NRT, and our results and positive outcomes were thus influenced by this treatment.

The long duration of the recruitment period (7 years) resulted from an adaptation of the frequency of ‘Day off’ interventions to the organizational possibilities of the Department of Psychiatry and the number of patients ready to commit to this intensive program, because most smokers were in pre-contemplation stages. However, no major changes that might have influenced smoking, such as hospital regulations (an indoor smoking ban) or forms of NRT used, were observed during this period.

### Limitations

Several limitations apply to this study. First, a control group was not included. Comparison with controls not attending the ‘Day off’ intervention as well as comparison of a group participating but not undergoing abstinence would be necessary to further study the effects of smoking withdrawal. Further, more intense monitoring, such as experience sample measurement, would provide additional information about the short-term evolution of craving. Another limitation is the short 26-hour time frame of analysis, because withdrawal symptoms change over time, and assessment after longer periods is necessary to study the effects after smoking cessation not only after temporary abstinence^7,52^. Tobacco withdrawal was observed to peak at 1 month, and short- and long-term symptoms differed^6^. The sample was insufficient for analyses of NRT use to elucidate characteristics of no-NRT users, because of the small number of subjects in this category. Interpretation was further limited by the absence of correction for confounding factors. For example, NRT use may be related to the strength of dependency, but sample size limits do not enable in depth investigation.

Finally we emphasize the complexity of smoking patterns, encompassing large interindividual differences leading to different withdrawal symptom patterns and abstinence attempt outcomes^53^. Most smokers report typical levels of withdrawal symptoms on cessation day, and more than one-third report extreme craving or negative affect or hunger after initial abstinence. The existence of different subgroups of smokers differing in tobacco-related, negative affect, sociodemographic, or outcome variables^53^ requires further research to clarify the associations between individual withdrawal profiles and psychopathology.

## CONCLUSIONS

Our results confirm the difficulty of assessing tobacco withdrawal in persons with mental health concerns. Interestingly, contrary to our expectations, short-term smoking related withdrawal symptoms did not increase but instead decreased in most psychiatric patients, who experienced abstinence in a stimulating and professional supportive context. Our data moderate the common assumption or myth that smoking abstinence leads to increased craving, particularly when NRT is available.

Craving, however, remained an interesting symptom to assess. It was the most prevalent withdrawal symptom, decreased more frequently among abstinent subjects, thus suggesting that craving might be a core symptom of smoking itself and may be an interesting predictor of cessation success, but only when measured during a cessation attempt.

This study highlighted that even patients with mental disorders can participate, and experience many benefits, in a motivational enhancement program based on a 26-hour tobacco abstinence experience.
